# Interstitial pneumonitis associated with pegylated interferon-alpha 2a and ribavirin for chronic hepatitis C infection: a case report

**DOI:** 10.1186/1757-1626-2-6464

**Published:** 2009-03-10

**Authors:** Ozlen Atug, Hakan Akin, Yesim Ozen Alahdab, Berrin Ceyhan, Nurdan Tozun, Osman Ozdogan

**Affiliations:** 1 Department of Gastroenterology, Marmara University School of Medicine, Istanbul, Turkey; 2 Department of Pulmonary Diseases, Marmara University School of Medicine, Istanbul, Turkey

## Abstract

Interstitial pneumonitis is a rare but potentially fatal side effect occurring from 2 weeks to 16 weeks after the initiation of treatment with pegylated interferon alpha and ribavirin for chronic hepatitis C.

Herein, we present a 68-year-old man with chronic hepatitis C virus infection who developed interstitial pneumonitis association with pegylated interferon after 36 weeks initiation of pegylated interferon-alpha and ribavirin therapy. He did not recover after discontinuation of pegylated interferon/ribavirin and improved by steroid therapy.

## Introduction

Chronic hepatitis C virus (HCV) infection is recognized as a global health problem with approximately 200 million people estimated to be infected worldwide. The treatment for chronic HCV infection has evolved over the last decade from standard interferon (IFN) monotherapy to combination therapy with standard IFN and ribavirin and more recently, pegylated (PEG) IFN and ribavirin combination therapy [1,2].

Common side effects of IFN include fatigue, flu-like symptoms, headache, arthralgia, myalgia, gastrointestinal, and neuropsychiatric symptoms. Rare serious adverse effects of IFN treatment are cardiac arrhythmias, cardiomyopathy, hearing loss, pancreatitis and pulmonary toxicity [3]-[6].

Reported pulmonary complications include interstitial pneumonitis, pulmonary sarcoidosis, pleural effusion, exacerbation of bronchial asthma and bronchiolitis obliterans organizing pneumonia [6].

Ribavirin is generally well tolerated; the major side effects of ribavirin are dose-dependent hemolytic anemia, cough, dyspnea, rash, depression, and dyspepsia. No cases of interstitial pneumonitis caused by ribavirin alone have yet been described to this date. We describe here a case of who developed interstitial pneumonitis following once weekly dose of PEG IFN-alpha in combination with ribavirin therapy for chronic HCV infection at 36 weeks after initiation of combination treatment and discussed the literature.

## Case presentation

A 68-year-old healthy man was evaluated in the ambulatory care facility for elevated liver enzymes. He was found to be asymptomatic and physical examination was unremarkable. Laboratory tests revealed an aspartate aminotransferase (AST) level of 72 U/L (normal up to 41 U/L), alanine aminotransferase (ALT) level of 89 U/L (normal up to 41 U/L), and positive serology for HCV. Serum electrolytes, complete blood count, and prothrombin time were normal. The HCV RNA titer by polymerase chain reaction was 200,000 copies/ml and genotype was 1b. Liver biopsy prior to PEG IFN *α*-2a/ribavirin therapy was denied by the patient. Due to persistent elevated ALT and AST for 6 months, therapy was started with 100 *µ*g of PEG IFN *α*-2a subcutaneously per week in combination with 800 mg ribavirin orally per day for chronic HCV infection. The patient responded well, his aminotransferase levels normalized, and HCV RNA was undetectable at week 12. The patient was continued combination therapy.

However, after 36 weeks of treatment, he presented with exertional dyspnea. Although full interferon dose was reinstituted, the dose of ribavirin was lowered to 600 mg/day because of anemia (Hb: 10.6 g/dl). Dyspnea progressed mild to moderate although the ribavirin dose was decreased.

**Figure 1:**
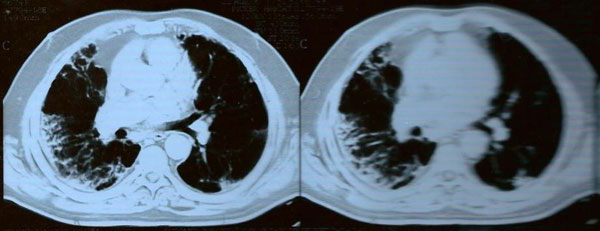
**High-resolution computed tomography of the chest (HRCT) demonstrates bilateral interstitial infiltrates, predominantly in the lung bases and periphery with subpleural parenchymal bands and ground-glass opacities that were greater on the right than on the left consistent with interstitial pneumonitis**.

Physical examination was remarkable for bilateral crackles at the lung bases. The patient was further treated with furosemide for presumed congestive heart failure but failed to improve. No third heart sound was audible, and there was no jugular venous distension, hepatojugular reflux, or other evidence of congestive heart failure. Echocardiography revealed normal left ventricular function. A chest x-ray performed before therapy was unremarkable. New chest x-ray and high-resolution computed tomography (HRCT) of the lung revealed bilateral interstitial infiltrates, predominantly in the lung bases and periphery with subpleural parenchymal bands and ground-glass opacities that were greater on the right than on the left consistent with interstitial pneumonitis (Figure 1). Pulmonary function tests (PFTs) revealed a restrictive defect and a diminished diffusing capacity. The forced expiratory volume in 1 second (FEV1) was 2.36 L (60% of predicted reference), the forced vital capacity (FVC) was 2.65 L (55% of predicted reference), and the FEV1/FVC percentage ratio was 89%. Diffusing lung capacity (DLCO) was 85% of predicted reference. Oxyhemoglobin saturation dropped to 87% while on breathing room air and the patient was consequently given oxygen therapy using a nasal cannula.

Interstitial pneumonitis secondary to PEG IFN *α*-2a and ribavirin was suspected. PEG IFN *α*-2a and ribavirin were discontinued at 36 weeks. HCV RNA by PCR was undetectable. One month after the discontinuation of treatment, symptomatic improvement was not noted. Therapy with 32 mg of methylprednisolone orally once per day was started by the consulting pulmonologist since mild exertional dyspnea and mild hypoxemia persisted.

The steroid dose was gradually tapered over the next several months and 4 mg/day methylprednisolone continued. Repeat PFTs showed marked improvement in his lung volumes and air flow.

**Figure 2:**
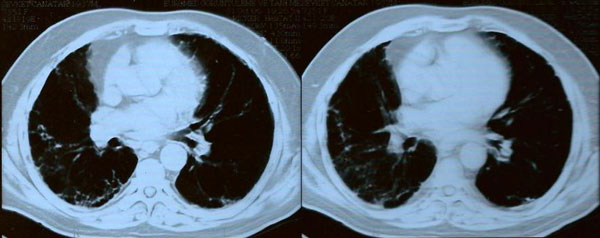
**Repeat HRCT of patient performed after two months discontinuation of PEG-IFN and ribavirin with systemic steroid therapy, showing improvement of the ground glass opacities**.

**Figure 3:**
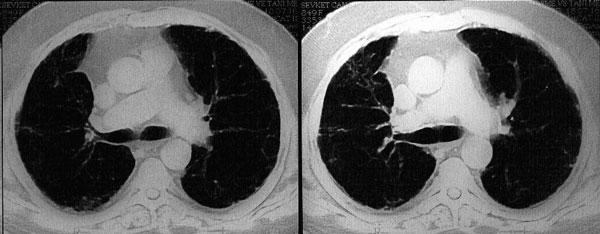
**Repeat HRCT of patient performed after eleven months discontinuation of PEG-IFN and ribavirin with systemic steroid showing resolution (improvement) of the ground glass opacities**.

Two months after cessation of therapy and with steroid treatment, a chest CT scan was repeated and compared to the previous CT scan. It showed marked improvement of the CT scan abnormalities but not complete resolution (Figure 2). Eleven months after the diagnosis of interstitial pneumonitis, steroid maintenance therapy was stopped by the consulting pulmonologist since the patient became asymptomatic with significant improvement in his chest CT scans (Figure 3).

HCV RNA by PCR was detectable 3 months after initiation of steroid therapy and increased to 4 x 10^6^ copy/ml one year after cessation of PEG IFN *α*-2a and ribavirin therapy.

## Discussion

Interstitial pneumonitis during PEG IFN alpha+ribavirin therapy for chronic hepatitis C patients is generally thought to be an unusual pulmonary side effect. There have been only 6 cases reported as a complication of the therapy with PEG-IFN alpha plus ribavirin for chronic HCV infection in the English literature [7]-[11]. Three of them died due to progressive hypoxemia and multisystem organ failure. These patients' death should be considered a drug-related mortality. To the best our knowledge, whilst the present case has been the 6th case of who developed interstitial pneumonitis during PEG IFN alpha+ribavirin therapy for chronic HCV infection, he has been third case as survived. Although in previously described 5 cases, interstitial pneumonitis usually developed from 2 weeks to 16 weeks after starting PEG IFN alpha/ribavirin therapy for HCV infection, our patient has also been the first case of who developed interstitial pneumonitis after 36 weeks of the treatment with PEG IFN alpha+ribavirin for chronic HCV infection in the literature. It suggested that interstitial pneumonitis can also develop at any time which either early or late during combination therapy with PEG IFN-alpha and ribavirin for chronic HCV infection.

In this patient, it seems highly likely that PEG IFN treatment was responsible for the onset of interstitial pneumonitis since all other possible infectious or systemic causes were ruled out, there existed a temporal relationship between the peg-interferon treatment and the onset of interstitial pneumonitis, the absence of any previous pulmonary history, and the specificity of the results of the various investigations carried out in the context of the interstitial pneumonitis. We used the Naranjo algorithm to show the probability of adverse drug reaction [12].

Interferon toxicity is generally dose dependent and increases with increasing dose and duration of treatment, although no clear relationship between dose and likelihood of side effects has been demonstrated [13]. PEG IFN provides higher levels of interferon because of longer absorption and prolonged half-life. Although the tolerability of PEG IFN alpha is comparable to that of conventional IFN alpha, pulmonary toxicity may occur more frequently with long-acting PEG IFN alpha therapy.

A potential role of ribavirin in pulmonary side effects during combination therapy with PEG IFN remains speculative. Ribavirin's known adverse symptoms of dyspnea and cough may suggest the possibility of independent pulmonary toxicity [14]. However, pulmonary side effects such as cough and dyspnea are common side effects and may make detection of interferon-related lung disease more difficult.

Ribavirin alone has never been reported as the cause for interstitial pneumonitis to this date; however, the rates of pulmonary toxicity with combination interferon/ribavirin seem no higher than with interferon alone. IFN-alpha seems to be the main agent for interstitial pneumonitis.

Although the pathogenesis associated with PEG IFN is not well known, two possible mechanisms have been put forward to explain drug-induced interstitial pneumonitis, one of which is the direct toxicity of the drug to the pulmonary organ, and the other is an indirect mechanism acting via immunological pathways. Most cases of interferon-associated pulmonary toxicity in patients with chronic hepatitis C reported in the literature were reversible, in many instances simply by discontinuation of therapy. Although some cases may respond well to discontinuation of interferon therapy, with or without corticosteroids, pulmonary involvement may not always be responsive [15]. There are only four reports of sustained interstitial pneumonitis despite withdrawal of IFN alpha /ribavirin and subsequent immunosuppressive therapy [8,9,11,15]. In fact, our patient has required low-dose steroid without complete resolution of CT scan abnormalities consistent with interstitial pneumonitis almost 1 year after cessation of combination therapy.

## Conclusion

This case described here suggests that the onset of disabling dyspnea in any time during PEG IFN-alpha and ribavirin therapy for chronic HCV infection should not be taken to be simply a pulmonary side effect of ribavirin. In clinical practice, the persistence of dyspnea after ribavirin dose adaptation should incite the physician to perform a pulmonary CT scan. Interstitial pneumonitis should be considered in the differential diagnosis and therapy must be withheld.

## Abbreviations

HCV: Chronic hepatitis C virus; IFN: Interferon; PEG IFN: Pegylated Interferon; AST: Aspartate aminotransferase; ALT: Alanine aminotransferase; HCV RNA: Hepatitis C Virus Ribonucleic Acid; Hb: Hemoglobin; HRCT: High-resolution computed tomography; PFTs: Pulmonary function tests; FEV1: Forced expiratory volume in 1 second; FVC: Forced vital capacity; DLCO: Diffusing lung capacity; CT: Computed tomography.

## Consent

Written informed consent was obtained from the patient for publication of this case report and accompanying images. A copy of the written consent is available for review by the Editor-in-Chief of this journal.

## Competing interests

The authors declare that they have no competing interests.

## Authors' contributions

OA and OO made substantial contributions to conception and design, and acquisition of data, and analysis and interpretation of data. HA interned the patient in the Internal Medicine Clinic and performed biochemical, hematological and radiological studies. YOA followed up the patient regarding the hepatological disease and a contributor in writing the manuscript. BC followed up and interpreted the patient regarding the pulmonological disease. NT involved in drafting the manuscript and revising it critically for important intellectual content and a contributor in writing the manuscript. OO was a major contributor in writing the manuscript. All authors read and approved the final manuscript. A copy of the written consent is available for review by the Editor-in-Chief of this journal.
